# *Leptospira* spp. Antibody Seroprevalence in Stray Dogs and Cats: A Study in Milan, Northern Italy

**DOI:** 10.3390/vetsci11100478

**Published:** 2024-10-05

**Authors:** Joel Filipe, Stefania Lauzi, Flavia Bullo, Mario D’Incau, Gabriele Meroni, Piera Anna Martino, Sonia Magistrelli, Maurizio Restelli, Paola Dall’Ara

**Affiliations:** 1 Department of Veterinary Medicine and Animal Health (DIVAS), University of Milan, Via dell’Università 6, 26900 Lodi, Italypaola.dallara@unimi.it (P.D.); 2 National Reference Centre for Animal Leptospirosis, Istituto Zooprofilattico Sperimentale della Lombardia ed Emilia Romagna “Bruno Ubertini”, Via A. Bianchi 1, 25121 Brescia, Italy; 3 One Health Unit, Department of Biomedical, Surgical and Dental Sciences (DISBIOC), University of Milan, Via Pascal 36, 20133 Milan, Italy; 4 Animal Health Service, Veterinary Department, ATS, 20122 Milano, Italy; 5 MSD Animal Health, Centro Direzionale Milano Due, Palazzo Canova, 20054 Segrate, Italy

**Keywords:** antibody titre, blood samples, microagglutination test (MAT), *Leptospira*, vaccination, serovars, serogroups, stray dog, stray cats, shelter

## Abstract

**Simple Summary:**

This study investigated the prevalence of *Leptospira* antibodies in stray dogs and cats in Milan, Italy. The results showed that 21.7% of the dogs tested seropositive for *Leptospira* antibodies, particularly the serovars *L.* icterohaemorrhagiae and *L.* Australis. In contrast, none of the cats tested seropositive. The study highlights the importance of ongoing serological surveillance in shelter environments to mitigate the zoonotic risk posed by leptospirosis.

**Abstract:**

Leptospirosis is a widespread zoonosis recognised as a re-emerging infectious disease in both humans and dogs, yet the actual seroprevalence of *Leptospira* in pets in Italy is relatively unknown. The aim of this study was to evaluate *Leptospira* antibody prevalence in dogs and cats from a shelter by the microscopic agglutination test (MAT), the gold standard test in leptospiral serology, and to assess risk factors for *Leptospira* infection. This seroepidemiological study investigated the prevalence of leptospiral antibodies in a cohort of 106 dogs and 51 cats housed in a municipal shelter in Milan. Blood samples were collected from the animals during two sampling periods: spring/summer 2014 and autumn/winter 2016/2017. Eight serogroups were evaluated: *L.* Australis, *L.* Ballum, *L.* Canicola, *L*. Grippotyphosa, *L.* Icterohaemorrhagiae, *L.* Pomona, *L*. Sejroe, and *L.* Tarassovi. Antibody titres ranged from 1:100 to 1:6400. The results indicated that 21.7% of dogs had antibodies against serogroups *L.* Icterohaemorrhagiae and *L.* Australis, making them the most often found. Conversely, none of the cats showed any presence of antibodies. Seropositivity was higher in the spring/summer period (32.7%) than in autumn/winter (11.1%), and no statistically significant results were found regarding sex or age. These findings underscore the importance of ongoing serological surveillance and biosecurity measures in shelter environments to mitigate the zoonotic risk posed by leptospirosis.

## 1. Introduction

*Leptospira*, members of the order *Spirochaetales* and family *Leptospiraceae*, are thin, spiral-shaped, Gram-negative bacteria responsible for leptospirosis, a zoonotic disease affecting both animals and humans, with transmission primarily through direct contact with infected animals or their fluids or environmental exposure [1]. Dogs are particularly vulnerable to *Leptospira* infection due to their frequent exposure to outdoor environments and water sources that may be contaminated by the bacteria [2]. They can contract leptospirosis through contact with infected water, soil, or other animals through urine, making them sick and/or potential disease carriers [3,4,5].

*Leptospira* infection in dogs is caused by various serovars belonging to different serogroups, including *L.* Canicola, *L.* Icterohaemorrhagiae, *L.* Grippotyphosa, *L.* Australis, *L*. Pomona, and *L*. Sejroe [3]. All serogroups are maintained by specific reservoir hosts, such as rats, mice, voles, and other small mammals [4,6]. In Italy, specific serovars such as *L.* Australis and *L.* Icterohaemorrhagiae have been identified in dogs with acute forms of the disease [7] and represent the most widespread and dangerous serovars in our country today [8]. In dogs, symptoms reflect the multisystemic nature of the disease and can range from fever, vomiting, diarrhoea, and dehydration to severe conditions such as systemic inflammation, liver dysfunction, pulmonary haemorrhage syndrome, acute kidney injury, and then chronic kidney disease. Treatment involves early administration of antibiotics to control the infection and manage clinical symptoms and fluid therapy for electrolyte imbalances [9,10,11,12].

While cats are traditionally considered less susceptible to *Leptospira* infection, recent studies suggest that they may act as reservoirs, playing a role in leptospirosis spreading, particularly in rural areas [13,14]. In some cases, they may become ill and show clinical symptoms, especially if they are already suffering from other diseases (e.g., panleukopenia, Feline Immunodeficiency Virus (FIV), or Feline Leukaemia Virus (FeLV) infections) [14,15,16,17,18].

The serogroups of *Leptospira* that generally affect cats include *L.* Australis, *L.* Icterohaemorrhagiae, *L.* Grippotyphosa, *L.* Pomona, and *L.* Sejroe [17,18,19]. These serogroups are known to be involved in incidental infections in felines, although the exact prevalence and impact of these infections vary based on geographical and environmental factors and are still not well studied [20,21]. In Italy, different studies have identified several serogroups affecting cats, including *L.* Icterohaemorrhagiae, *L.* Grippotyphosa, *L.* Pomona, and *L*. Ballum [17,19]. A recent study highlighted the presence of serogroup *L.* Australis in cats, marking a significant finding in the European context [18].

In humans, leptospirosis can be present in several anicteric or icteric (Weil’s syndrome) forms, leading to a variety of mild to severe symptoms, including flu-like syndrome, jaundice, red eyes, rash, and organ failure [22]. Human leptospirosis cases in Europe have been documented in various countries. Croatia reported one of the highest incidences of human leptospirosis in Europe in 2010, with an annual incidence rate of 1.83/100,000 human population [23] and 1.53/100,000 human population in the period 2009–2014 [24]. Portugal also has a significant rate of the disease, ranking third among European countries with the highest rate per 100,000 population in 2016 (particularly in rural areas) [25], and 0.50 cases/100,000 population in 2022. In the 2024 EU/EEA (European Union/European Economic Area) report analysing human leptospirosis cases in the period 2010 to 2021, 23 countries have reported 12,180 confirmed cases of the disease (annual mean of 0.24 cases/100,000 population), especially in Germany, France, Netherlands, Romania, and Portugal, all these accounting for 79% of all cases. Slovenia held the highest rate of notifications (0.82 cases/100,000 population). Globally, from 2010 to 2021, the reporting rate increased by 5.0% per year despite the COVID-19 pandemic and the related behavioural changes [26]. Finally, in the last available ECDC (European Centre for Disease Prevention and Control) annual epidemiological report dated June 2024, 765 cases of confirmed leptospirosis (0.18 cases/100,000 human population) were reported in the EU/EEA in 2022, and France had the highest number of confirmed cases (245), with an annual mean of 0.36 cases/100,000 human population [27].

The diagnostic methods for canine leptospirosis include PCR and the microagglutination test (MAT), and various serological assays such as enzyme-linked immunosorbent assays (ELISAs) and rapid immunochromatographic tests looking for specific IgG and/or IgM antibodies [28,29,30,31,32,33]. PCR is a highly sensitive method used in the early diagnosis of the disease, allowing for the rapid detection of *Leptospira* DNA in blood or urine samples. However, MAT (considered the gold standard) is a serological test that detects specific antibodies to identify the *Leptospira* serogroup [28,34,35]. Vaccination has significantly reduced the prevalence of certain serovars that belong to different serogroups worldwide, specifically in dogs, and it remains a key preventive measure, with various available vaccines targeting different serogroups to control infection and urinary shedding [9,11,36].

Due to their lifestyle and exposure to various environments, stray dogs and cats play a crucial role in the epidemiology of *Leptospira* infections. Knowing the seroprevalence of *Leptospira* spp. in these populations is essential to understand the exposure and potential circulation of *Leptospira* among them [9,10,11,37,38,39]. Different studies have shown that stray and shelter dogs have a higher prevalence of *Leptospira* infection than pet dogs, highlighting the importance of investigating stray animal populations [2,39,40,41]. Additionally, investigations in various regions around the globe have revealed significant seroprevalence rates of *Leptospira* in stray dogs and sometimes also in stray cats, emphasising the global relevance of studying these populations [37,42,43,44,45]. Furthermore, *Leptospira* infection in stray animals poses a potential risk for human transmission, especially in areas where they can interact with wild rodents, which can act as reservoirs for the bacteria [45,46].

Given the public health implications of leptospirosis, particularly within environments where stray animals may meet humans, this study aims to assess the exposure to Leptospira of dogs and cats housed in a municipal shelter in Milan (Italy). The study also seeks to identify the most prevalent serogroups in the dog population and assess the potential role of cats as reservoirs of the bacteria.

## 2. Materials and Methods

### 2.1. Animals

A total of 106 dogs and 51 cats housed at the municipal shelter in Milan were included in this seroepidemiological investigation. The animals were admitted to the facility during two sampling periods: spring/summer 2014 (52 dogs and 51 cats) and autumn/winter 2016/2017 (54 dogs). The study cohort represented a diverse population in terms of age, breed, and origin, reflecting the typical composition of animals in shelter environments. All animals were generally healthy, with only a few exhibiting signs of undernourishment and dehydration. However, none displayed symptoms of leptospirosis.

The Milan municipal dog shelter is managed by the ATS (Agenzia di Tutela della Salute) of the metropolitan city of Milan and is responsible for urban health and animal health services, including rabies prophylaxis and stray animal control. The shelter handles various animals, including strays, injured or sick animals, and cats from colonies for mandatory sterilisation. It also conducts clinical and behavioural assessments and carries out necessary medical and preventative care, such as vaccinations and antiparasitic treatments.

### 2.2. Sample Collection

Samples were collected from the shelter dogs and cats for diagnostic purposes or routine health checks. Leftover specimens were used in this study. Thus, a formal approval of the Ethical Committee was not required based on the guidelines of the investigators’ institution (EC decision, 29 October 2012, renewed with the protocol n° 02-2016).

Blood samples were aseptically collected during routine medical examinations from each animal via venipuncture of the cephalic or jugular vein, and then centrifuged at 1500× *g* for 15 min. The resulting serum (±0.5 mL) was stored in labelled Eppendorf tubes (Eppendorf AG, Hamburg, Germany) and frozen at −20 °C until serological analysis.

Additionally, the study considered cats, most of them colony ones (and thus temporarily housed in the shelter for mandatory sterilisation) and the remainder permanently housed at the shelter. Blood samples were collected, and sera were stored using the same procedures as for the dogs.

### 2.3. Microagglutination Test (MAT)

Serum samples were subjected to serological testing for leptospiral antibodies using MAT. The serum samples were analysed by the Bacteriology Unit of Istituto Zooprofilattico Sperimentale della Lombardia e dell’Emilia-Romagna (Brescia, Italy). The antigen panel was composed of 8 serogroups/serovars: *L.* Australis/Bratislava, *L.* Ballum/Ballum, *L.* Grippotyphosa/Grippotyphosa, *L.* Canicola/Canicola, *L.* Pomona/Pomona, *L.* Sejroe/Hardjo, *L.* Tarassovi/Tarassovi, and *L.* Icterohaemorrhagiae/Copenhageni. The method followed WOAH (World Organization for Animal Health) guidelines. Briefly, sera were diluted from 1:100 to 1:16.400 in pH 7.2 phosphate buffer. Dilutions were executed with microtitre 96-well ELISA plates: 25 µL serum sample was incubated with 25 µL panel antigen for 1 h at 37 °C. Then, the result was analysed using a dark-field optical microscope. For the interpretation of seropositivity at different titres, we used the cut-off of 1:400 as in the study of Tagliabue et al. (2016) [47], which was crucial in distinguishing between vaccination-induced antibodies and those from field exposure. Antibody titres ≥ 1:100 were considered seropositive, while an antibody titre ≥ 1:400 was considered seropositive after field exposure.

### 2.4. Statistical Analysis

Descriptive statistics were used to summarise the demographic features of the animals, including age, sex, and breed distribution. Serogroup-specific antibody titres were recorded, and the prevalence of different serovars was calculated.

Statistical analyses were conducted using GraphPad Prism version 9. The Chi-square test of independence was employed to determine whether there was a significant association between the categorical variables under study. This test was chosen to evaluate the relationships between the seropositivity to leptospirosis and other variables, such as sex and age categories.

## 3. Results

### 3.1. Dog Samples

Of the 106 tested dogs, 23 (21.7%) were serologically positive for one or more serogroups (Table 1). The highest positivity rates were observed for the serogroups *L.* Australis, *L*. Canicola, *L.* Grippotyphosa, *L.* Icterohaemorrhagiae, and *L.* Pomona, with titres ranging from 1:100 to 1:6400 (Table 2). No sample was seropositive for *L.* Ballum, *L.* Sejroe, or *L*. Tarassovi.

Among the seropositive samples, 52.2% showed seropositivity to a single serogroup (26.1% to *L.* Icterohaemorrhagiae, 17.4% to *L.* Canicola, 4.3% to *L.* Australis, and 4.3% to *L.* Grippotyphosa).

Moreover, 43% of samples exhibited seropositivity to two serogroups (21.7% to *L.* Australis and *L.* Icterohaemorrhagiae; 8.7% to *L.* Canicola, and *L.* Icterohaemorrhagiae; 4.3% to *L.* Grippotyphosa and *L.* Pomona; and 4.3% to *L.* Icterohaemorrhagiae and *L.* Grippotyphosa).

Only one sample demonstrated seropositivity to three serogroups (*L.* Australis, *L.* Canicola, and *L.* Icterohaemorrhagiae).

#### 3.1.1. Dogs Sampled during Spring/Summer 2014

Among the 52 dogs sampled during the 2014 spring/summer period, 17 (32.7%) were seropositive. The highest seropositivity rates were observed for the serogroups *L.* Australis, *L.* Canicola, and *L.* Icterohaemorrhagiae, with titres ranging from 1:100 to 1:6400 (Table 2).

Of the 17 seropositive samples, 52.9% exhibited seropositivity to two serogroups (six samples to *L.* Australis and *L.* Icterohaemorrhagiae, 11.8% to *L*. Canicola and *L.* Icterohaemorrhagiae, and 5.9% to *L.* Grippotyphosa and *L.* Pomona), 41.2% to a single serogroup (23.5% to *L.* Canicola and 17.6% to *L.* Icterohaemorrhagiae), and one to three serogroups (*L.* Australis, *L.* Canicola, and *L.* Icterohaemorrhagiae).

#### 3.1.2. Dogs Sampled during Autumn/Winter 2016/2017

Among the 54 dogs sampled during the 2016/2017 autumn/winter period, 7 (13%) were seropositive. The highest positivity rates were observed for the serogroups *L.* Australis, *L.* Grippotyphosa, and *L.* Icterohaemorrhagiae, with titres ranging from 1:100 to 1:800 (Table 2), significantly lower than those observed in the spring/summer period (*p*-value = 0.0067).

Of the seven seropositive samples, 71.4% exhibited seropositivity to a single serogroup (42.9% to *L*. Icterohaemorrhagiae, 14.3% to *L.* Australis, and 14.3% to *L.* Grippotyphosa), and 14.3% to two serogroups (*L.* Grippotyphosa and *L.* Icterohaemorrhagiae).

#### 3.1.3. Comparison between the Two Sampling Seasons

Comparing the two sampling seasons, a reduction in seropositivity from the 2014 spring/summer period to the 2016/2017 autumn/winter period was noted (17 seropositive samples in 2014 versus 6 in 2016/2017), with seropositivity decreasing from more than 30% in the first period to about 11% in the second one.

In both periods, *L.* Icterohaemorrhagiae was the most frequently involved serogroup, consistently showing the highest seroprevalence in dogs. Notably, seropositivity to *L.* Canicola, detected in seven samples in 2014, was no longer observed in any sample of the 2016/2017 period (Table 1).

#### 3.1.4. Analysis of Seropositivity by Sex

Of the 23 seropositive subjects, 12 (52.2%) were females (9 sexually intact and 3 neutered), and 11 (47.8%) were males (10 sexually intact and 1 neutered). Statistically, seropositivity was not correlated with sex (*p*-value = 0.644). Considering the two periods separately, the percentage of positivity remained around 50% for each sex: in 2014, nine females and eight males were seropositive, while in 2016–2017, three females and three males were seropositive.

#### 3.1.5. Analysis of Seropositivity by Age

Among the seropositive dogs, 4 were puppies (less than 1 year old), 16 were adults, and 3 were senior/geriatric. The highest positivity was observed in adults, but no particular relationship existed with a specific age group. Considering the two periods separately, in 2014 positivity was found in three puppies and two senior/geriatric dogs, while the other seropositive dogs were aged between 1 and 7 years. In 2016–2017, positivity was found in one puppy and one senior/geriatric dog, while the other seropositive dogs were aged between 1 and 3 years. No significant statistical association with age was observed (*p*-value = 0.923).

### 3.2. Cat Samples

Unlike the dog samples, all 51 cat samples tested negative in the microagglutination test.

## 4. Discussion

Leptospirosis is a re-emerging, life-threatening zoonotic disease present worldwide, especially where heavy flooding and rainfall are common. The disease is transmitted by contact with water or soil contaminated by the urine of potentially infected farm or wildlife animals (especially rodents). According to the latest findings shared by the scientific community, all dogs must be considered at risk of leptospirosis, regardless of geographic location, lifestyle, breed, age, or seasonal time [9,11]. This is why the new WSAVA (World Small Animal Veterinary Association) 2024 guidelines have emphasised that in countries where the disease is endemic, the serogroups responsible are known, and valid vaccines are available, vaccination for leptospirosis should be considered core, and is highly recommended for all dogs [47].

The seroepidemiological investigation conducted in this study sheds light on the prevalence of *Leptospira* antibodies in dogs and cats housed at the municipal shelter in Milan. The detection of leptospiral antibodies in a significant proportion of the sampled population asymptomatic of leptospirosis (more than 20% of dogs) underscores the potential exposure of these animals to *Leptospira* spp., highlighting the importance of understanding the dynamics of this zoonotic disease in companion animals.

The interpretation of seropositivity at different titres, using the cut-off of 1:400 as used in the study of Tagliabue et al. [48], was crucial in distinguishing between vaccination-induced antibodies and those from field exposure. Even if no information about the vaccination status of the dogs was available, samples with titres between 1:100 and 1:400 likely indicated vaccination, particularly when matching the serogroups in commercially available vaccines, with some exceptions. For example, one dog had a titre of 1:100 for *L.* Pomona, not included in any commercially available vaccine in Italy, and this may suggest a potential exposure to this specific serogroup in the field. Samples with titres of 1:400, a value considered by many authors as the threshold for indicating infection rather than vaccination, posed challenges in interpretation when related to serovars also present in vaccines. Conversely, the higher titres (between 1:800 and 1:6400) found in five animals probably indicated a field exposure, despite the absence of clinical symptoms at the sampling time.

These findings underscore the complexity of differentiating between vaccination-induced antibodies and antibodies due to actual field exposure, especially when considering specific titres and serogroups. For this reason, some authors prefer to consider 1:800 as the threshold value for discriminating between field infection antibodies and vaccine antibodies, and the analysis of paired (acute and convalescent) samples using MAT as the reference standard test for diagnosis of leptospirosis [9,11,49]. The lack of complete medical histories for the dogs arriving at the shelter further complicates the definitive determination of the origin of the seropositivity.

It should also be noted that the dogs involved in this study are all from shelters, and therefore their vaccination history is unknown. Additionally, in 2014–2017, vaccination against leptospirosis was not given the same importance as it is today, meaning that only a few dogs were vaccinated against this dangerous disease. Consequently, any positivity to vaccine serovariants could still be linked to field infections rather than vaccination. This is especially true for large, male, adult dogs (that were used in hunting). Owned dogs were not normally vaccinated because the risk of contact with the pathogen was considered low and its maintenance hosts were not known, as they are today, when maintenance hosts have also become much more urbanised.

This study considered two sampling periods corresponding to different seasons, spring/summer and autumn/winter, to assess any seasonal variations in leptospirosis prevalence among the dogs. Of the animals sampled in spring/summer 2014, 32.7% were seropositive, while when considering the dogs sampled during the 2016/2017 autumn/winter period, only 11.1% were seropositive, representing a reduction of 21.6% from the previous sampling. When considering the total number of dogs, the samples from the spring/summer period were more numerous and with a higher seropositivity to different serogroup frequencies than those of the autumn/winter period. This lower seropositivity is probably due to unfavourable climatic factors that may have reduced the possibility of reservoir dissemination (mainly by mice and rats) and consequently direct contact with them. Seasonal variation in canine leptospirosis has already been documented, and seropositive cases normally increase from late summer to early autumn [50]. During the summer, an increase in leptospirosis cases has been reported, especially in northern temperate zones [51]. Seasonal changes, environmental factors, and regional variations may play significant roles in the prevalence and transmission of leptospirosis in dogs. In our study, the decline in seropositivity observed over time could be attributed to both the different seasonality (a warm and humid climate increases the likelihood of pathogen dissemination) and a possible improvement in the epidemiological situation related to the availability of valid quadrivalent (L4) vaccines. It should be emphasised that L4 vaccines (containing not only *L.* Canicola and *L.* Icterohaemorrhagiae but also *L.* Grippotyphosa and *L.* Australis) have only been marketed in Italy since the second half of 2014. Consequently, any positivity for *L.* Grippotyphosa and *L.* Australis detected in the spring/summer 2014 sampling is likely related to field infections rather than vaccination.

It should not be forgotten that the two samples were taken not only in different seasons but also in different years; as a result, the decline in the results obtained could be related to a different seroprevalence in that particular year. This could represent a limitation of the study, as it does not allow for the evaluation of the effect of a single season.

When taking into consideration variables like sex or age, no statistically significant results were found in the populations. However, some studies indicate that male dogs are generally at a higher risk for leptospirosis compared to females. For instance, a study conducted in Jakarta found that 80% of the dogs diagnosed with leptospirosis were male, while only 20% were female [52]. This aligns with findings from other studies, which suggest that male dogs, particularly those involved in outdoor activities, are more likely to be exposed to the bacteria due to behaviours such as sniffing and licking contaminated surfaces [53,54]. Conversely, other studies have reported a higher prevalence of leptospiral infections in female dogs, suggesting that environmental factors and lifestyle surely influence these outcomes [55,56].

Age seems to be a critical factor that may also influence the susceptibility of dogs to leptospirosis, but while some studies have shown that older dogs are at a greater risk of infection than younger ones [54], other studies demonstrated that those younger than 1 year old have a significantly higher risk of infection compared to older ones [43,53].

Seropositive samples were found all around the Milan hinterland. However, interestingly the presence of two seropositive dogs was observed in the same zone (“Lampugnano”) in two different years (2014 and 2017) but for different leptospiral serogroups (*L.* Canicola in 2014 and *L.* Grippotyphosa in 2017). Lampugnano is a particularly green area in the northwest of Milan, hosting different rodent species (e.g., mice, rats, and coypus) that can serve as reservoirs for *Leptospira* spp. Therefore, it can be assumed that this is a potentially high-risk area for *Leptospira* spp. infection for the wild and domestic animals that frequent it.

Identifying antibody positivity for specific serogroups provides valuable insights into the epidemiology of leptospirosis in the shelter environment [5,57]. The diversity of serovar positivity underscores the complex nature of *Leptospira* strains circulating among dogs, necessitating a multifaceted approach to vaccination strategies and disease control [58,59]. Knowing the type and distribution of circulating serogroups can help implement targeted interventions to reduce the burden of leptospirosis in both animal and human populations, remembering of course that while seroepidemiological studies are important, the genomic characterisation of local strains confirms what is actually circulating.

Due to the low numerosity of our sampling, the results of this study cannot exclude the seropositivity of cats for *Leptospira* spp. in the territory of the municipality of Milan. Several studies, especially some recent ones, point to the cat as a possible host of different leptospiral serogroups, even if clinical cases of feline leptospirosis are really rare [14,16,17,18,60].

Cats can develop antibodies against *Leptospira* spp. sometimes playing a significant role in the epidemiology of leptospirosis [42,61]. Some studies have shown that cats can carry and shed pathogenic *Leptospira* spp. in their urine for extended periods after infection, indicating their potential role in transmitting the disease [16,42].

Cats with antibodies against *Leptospira* serogroups may rarely exhibit signs associated with kidney disease, highlighting the potential impact of the bacteria on cats’ renal health [60,62]. Despite the low prevalence of clinical leptospirosis in cats, outdoor cats have been found to have a higher seroprevalence compared to indoor ones, emphasising the importance of environmental exposure in disease transmission [63]. Furthermore, cats dwelling in environments with livestock, such as dairy farms, can be implicated in the epidemiology of *Leptospira* infection, suggesting interspecies transmission between livestock and domestic cats [64]. Although the treatment of leptospirosis in cats is debated and not accepted by all, it is essential to consider the potential risks of the *Leptospira* infection and their possible impacts on cat welfare, too [65].

In a study conducted in Sicily, Italy, serological and molecular evidence indicated the presence of *L.* Icterohaemorrhagiae in stray dogs and cats, suggesting that these animals may serve as reservoirs for the pathogen [19]. This finding aligns with previous reports that identified *L.* Icterohaemorrhagiae as a prevalent serogroup in dogs [17,19].

Furthermore, in 2022 Balboni et al. reported an outbreak of *L.* Sejroe in a kennel, highlighting the importance of dogs as sentinels for leptospirosis in specific environments [5]. This study underscores the role of domestic dogs in the epidemiology of leptospirosis, particularly in kennel settings where close contact among animals can lead to increased transmission rates. The identification of serogroups such as *L.* Australis and *L.* Icterohaemorrhagiae in symptomatic dogs further illustrates the diversity of serogroups present in the Italian canine population [66].

In cats, the prevalence of leptospirosis appears to be less well documented, but emerging evidence suggests that they are also susceptible to infection. A recent study reported the presence of *L.* Australis in outdoor cats. Interestingly, the study found that cats living near flood-prone areas had a significantly higher likelihood of seropositivity, similar to findings in dogs [20]. This suggests that environmental exposure is a critical factor influencing leptospirosis prevalence in both species.

The observed seroprevalence in the dogs of this study also aligns with previous studies reporting a high prevalence of leptospiral antibodies in dogs and emphasising the zoonotic nature of the disease [67,68]. The results underscore the need for strong monitoring systems and preventative measures to control the spread of leptospirosis from animals to people, particularly in densely populated metropolitan areas where pets play a vital role in transmitting the disease.

Implementing stringent biosafety protocols within shelters is crucial in preventing the introduction and spread of leptospirosis among resident animals, while minimising the risk of transmission to humans [69,70]. Different measures, including routine disinfection practices, strict control of animal movement, and proper waste management, are essential components of comprehensive disease prevention strategies aimed at safeguarding both animal and human health.

This study has the potential to provide useful insights for establishing specific approaches to manage and prevent *Leptospira* spp. infection by understanding the exposure levels and serovar distribution among shelter animals.

## 5. Conclusions

Leptospirosis is a serious and sometimes fatal disease of animals and humans that can and should be controlled with appropriate measures (e.g., prompt diagnosis, good hygiene, and vaccination) to be applied constantly as early as possible to limit its spread in maintenance and accidental hosts. This study contributes valuable data on the seroepidemiology of leptospirosis in dogs and cats residing in a municipal shelter environment. By elucidating the seroprevalence and serovar diversity of *Leptospira* spp., this research enhances our understanding of the epidemiological landscape of leptospirosis in companion animals, especially dogs. Thus, assessing *Leptospira* exposure and serovar prevalence in shelter animals is a critical biosecurity tool in managing human and animal health risks. Regular monitoring and surveillance are paramount to managing and reducing zoonotic risks in these species. The results of this study underscore the importance of continued surveillance, targeted control measures, and public health interventions to mitigate the zoonotic risk associated with leptospirosis transmission from animals to humans.

## Figures and Tables

**Table 1:** Number of samples seropositive for different serogroups in the two different periods considered.

	*L.* Australis	*L.* Ballum	*L.* Canicola	*L.* Grippotyphosa	*L.* Icterohaemorrhagiae	*L.* Pomona	*L.* Sejroe	*L.* Tarassovi	Total
Spring/summer	8	0	7	1	11	1	0	0	28
Autumn/winter	1	0	0	2	4	0	0	0	7
Total	9	0	7	3	15	1	0	0	

**Table 2:** Seropositive samples in the two different periods considered divided by antibody titres.

	Antibody Titres					
	1:100	1:200	1:400	1:800	1:6400	Total
Spring/summer	10	7	5	5	1	28
Autumn/winter	1	3	1	2	0	7
Total	11	10	6	7	1	

## Data Availability

All the data are available within the article and from the corresponding author upon reasonable request.
